# A Two-Year Review on Epidemiology and Clinical Characteristics of Dengue Deaths in Malaysia, 2013-2014

**DOI:** 10.1371/journal.pntd.0004575

**Published:** 2016-05-20

**Authors:** Yuan Liang Woon, Chee Peng Hor, Narwani Hussin, Ariza Zakaria, Pik Pin Goh, Wee Kooi Cheah

**Affiliations:** 1 Clinical Epidemiology Unit, National Clinical Research Centre, Kuala Lumpur, Malaysia; 2 Kepala Batas Hospital, Kepala Batas, Penang, Malaysia; 3 Clinical Research Centre, Seberang Jaya Hospital, Seberang Jaya, Penang, Malaysia; 4 Clinical Research Centre, Taiping Hospital, Perak, Malaysia; 5 National Clinical Research Centre, Kuala Lumpur, Malaysia; 6 Department of Medicine, Taiping Hospital, Perak, Malaysia; Duke-NUS Graduate Medical School, SINGAPORE

## Abstract

**Background:**

Dengue infection is the fastest spreading mosquito-borne viral disease, which affects people living in the tropical and subtropical countries. Malaysia had large dengue outbreaks in recent years. We aimed to study the demographics and clinical characteristics associated with dengue deaths in Malaysia.

**Methods:**

We conducted a retrospective review on all dengue deaths that occurred nationwide between 1^st^ January 2013 and 31^st^ December 2014. Relevant data were extracted from mortality review reports and investigational forms. These cases were categorized into children (<15 years), adults (15–59 years) and elderly (≥60 years) to compare their clinical characteristics.

**Results:**

A total of 322 dengue deaths were reviewed. Their mean age was 40.7±19.30 years, half were females and 72.5% were adults. The median durations of first medical contact, and hospitalization were 1 and 3 days, respectively. Diabetes and hypertension were common co-morbidities among adults and elderly. The most common warning signs reported were lethargy and vomiting, with lethargy (p = 0.038) being more common in children, while abdominal pain was observed more often in the adults (p = 0.040). But 22.4% did not have any warning signs. Only 34% were suspected of dengue illness at their initial presentation. More adults developed severe plasma leakage (p = 0.018). More than half (54%) suffered from multi-organ involvement, and 20.2% were free from any organ involvement. Dengue deaths occurred at the median of 3 days post-admission. Dengue shock syndrome (DSS) contributed to more than 70% of dengue deaths, followed by severe organ involvement (69%) and severe bleeding (29.7%).

**Conclusion:**

In Malaysia, dengue deaths occurred primarily in adult patients. DSS was the leading cause of death, regardless of age groups. The atypical presentation and dynamic progression of severe dengue in this cohort prompts early recognition and aggressive intervention to prevent deaths.

**Trial Registration:**

National Medical Research Registry (NMRR, NMRR-14-1374-23352)

## Author Summary

Dengue infection, especially severe dengue, affected more of adults from working age groups in the society. They can present with non-specific symptoms mimicking many other febrile illnesses, or severe symptoms suggestive of sepsis, with low suspicion of dengue. The clinical progression in severe dengue can be dynamic and sometimes unanticipated, whereby patients can deteriorate rapidly in a short period of time and succumb to death. Although children tend to have central nervous system involvement, where they presented with confusion and/ or seizure, and more elderly had heart involvement, the primary cause of dengue death, dengue shock syndrome, did not differ across different age groups.

## Introduction

Dengue infection has been identified as the fastest spreading mosquito-borne viral disease by World Health Organization (WHO) [1]. At least 3.6 billion people living in the subtropical and tropical regions are at risk of contracting dengue, imposing substantial socioeconomic impact to affected populations [2].

Since the first outbreak in Malaysia in 1902, dengue has become a notifiable infection by 1973 [3]. Malaysia is endemic with all four dengue virus serotypes co-circulating over the past two decades [4], with predominantly *DENV1* and *DENV2* responsible for the escalating number of cases over the recent years [5]. Major outbreaks occurred in a cyclical pattern of approximately 8 years [6]. However, the reported cases had increased from 7,103 in 2000 to 43,346 in 2013, with a drastic escalation to 108,698 in 2014 [5]. While dengue mortality increased over the years, the case fatality ratio remained between 0.16% and 0.30% over the past decade [5]. Studies have revealed more severe manifestations with multisystem involvement for dengue infections, and an epidemic shift of age range incidence from children to adults in Malaysia [3, 4].

To date, there is no cure for dengue infections, apart from supportive treatments. There have been attempts to look at the risk factors associated with dengue haemorrhagic fever and dengue shock syndrome (DSS) focusing on dengue patients in single tertiary centres [7, 8]. Local published data on epidemiological characterisation and clinical spectrum of the fatal dengue cases remain limited. This review aims to study the epidemiology, clinical characteristics and cause of deaths on fatal dengue cases nationwide for 2013 and 2014.

## Methods

### Study setting and populations

This was a retrospective observational study reviewing all the dengue mortality cases which occurred in Malaysia between 1^st^ January 2013 and 31^st^ December 2014. Notification of all dengue deaths in Malaysia are mandatory. All cases were hospitalized and deaths occurred in hospital setting. Doctors managing each case were required to complete and submit the dengue specific investigational forms to the Ministry of Health. All these cases were reviewed by a group of independent consultants across different specialties at dengue mortality meetings held by each state health of departments. All relevant details pertaining to patient’s initial presentation and diagnosis, course of management in relation to the dynamic disease progression, and subsequent cause of death were reviewed extensively together with the managing teams during the meeting. It was aimed to identify areas for improvement and strengthen patients’ care to reduce mortality in future cases, apart from educational purpose. Detailed discussion was then documented in mortality review report.

The lists of patients’ names were retrieved from the Vector Borne Disease Control Division, Ministry of Health, Malaysia. Predesigned case report form (S1 Fig) was employed to collect all basic demographics, pre-existing co-morbidities, clinical and outcome data from the source documents, which included all mandatory investigational forms as well as mortality review reports from hospitals and state health departments, whichever available.

### Definition of epidemiological and clinical variables

All cases were categorized into three age groups, namely children aged <15 years, adults aged 15–59 years, and elderly aged ≥60 years. Two cases with missing age were excluded from subsequent analyses involving comparison across the different age groups.

The case definition for dengue infection, definitions of warning signs, severe dengue and dengue shock syndrome (DSS) were referred to the WHO 2009 guideline [9]. All cases were confirmed seropositive for dengue non-structural glycoprotein 1 (NS1), and/or Immunoglobulin M (IgM) antibodies, and/or by polymerase chain reaction (PCR). The presence of NS1 antigen, IgM and IgG antibodies in the sera was detected either using the dengue-specific combinational rapid test (Standard Diagnostics BIOLINE Dengue Duo, Korea) or enzyme-linked immunosorbent assay (ELISA) test available at local hospitals, or samples were sent to the National Public Health Laboratory in Selangor to proceed with ELISA test if neither test was available in the hospital.

For the classification of various organ involvement in this cohort, liver involvement refers to high titre of alanine aminotransferase and/ or aspartate aminotransferase beyond 1000 iu/L, or acute liver failure. Kidney involvement refers to acute renal failure or acute exacerbation on chronic kidney disease. Heart involvement refers to myocarditis, pericarditis and/ or heart failure, either acute onset or exacerbation of the underlying heart disease, while central nervous system involvement refers to encephalopathy, encephalitis as well as intracranial bleeding.

### Ethical approval

This study was registered under National Medical Research Registry (NMRR, NMRR-14-1374-23352) and approved by Medical Research Ethics Committee, Ministry of Health.

### Statistical analysis

Data were compiled and analysed using Statistical Package for Social Sciences program version 21 (SPSS: Inc. Chicago. Il. USA). All data were anonymized during analysis. Categorical variables were expressed in frequency and proportion. The normally distributed continuous variables were reported in means ± standard deviation, while the non-normally distributed continuous variables were reported in medians (25^th^ percentile, 75^th^ percentile). Comparisons across different age groups for initial presentation with warning signs, pre-morbid illnesses, criteria for severe dengue and causes of death were performed using Pearson Chi-Square tests. The two-sided statistical significance level, p-value, was set at 0.05 for all inferential analyses in this study.

## Results

### Demographic profiles

This study included 322 fatal dengue cases, with 94 in 2013 and 228 in 2014. The mean age of this cohort was 40.7 ± 19.30 years. One in ten of the cases were children aged <15 years while 17.5% were elderly aged 60 years and beyond (Table 1). The youngest case was a 10-day old female infant who died of DSS while the eldest was a 90-year old lady with multiple co-morbidities who died of severe dengue with upper gastrointestinal bleeding. Majority were adults from the working age group. Six of them were pregnant women and succumbed due to DSS. Half of our cohort (51.2%) were females. More than half of them (54%) were of Malay ethnic, followed by Chinese (21.7%), Indian (11.2%) and other minor ethnics (4.7%), reflecting closely the ethnic distribution in the country. Up to 8.1% of the deceased persons were of foreign nationalities, with 14 Bangladeshi, three Indonesians, three Nepalese, three Burmese, two Thai and one Somalian.

**Table 1:** Demographic Profile of Fatal Dengue Cases in Malaysia.

Demographics	Children (<15 years) *n* (%)	Adults (15–59 years) *n* (%)	Elderly (≥60 years) *n* (%)
Cases (per overall)	32 (10)	232 (72.5)	56 (17.5)
Mean age (in years)	6.8 ± 4.54	38.5 ± 11.64	69.2 ± 7.90
Gender			
Male	16 (50)	109 (47)	30 (53.6)
Female	16 (50)	123 (53)	26 (46.4)
Ethnicity			
Malay	20 (62.5)	124 (53.4)	28 (50)
Chinese	3 (9.4)	47 (20.3)	20 (35.7)
Indian	4 (12.5)	28 (12.1)	4 (7.1)
Other minor ethnics	3 (9.4)	9 (3.9)	4 (7.1)
Foreign nationals	2 (6.2)	24 (10.3)	0

**Fig 1:**
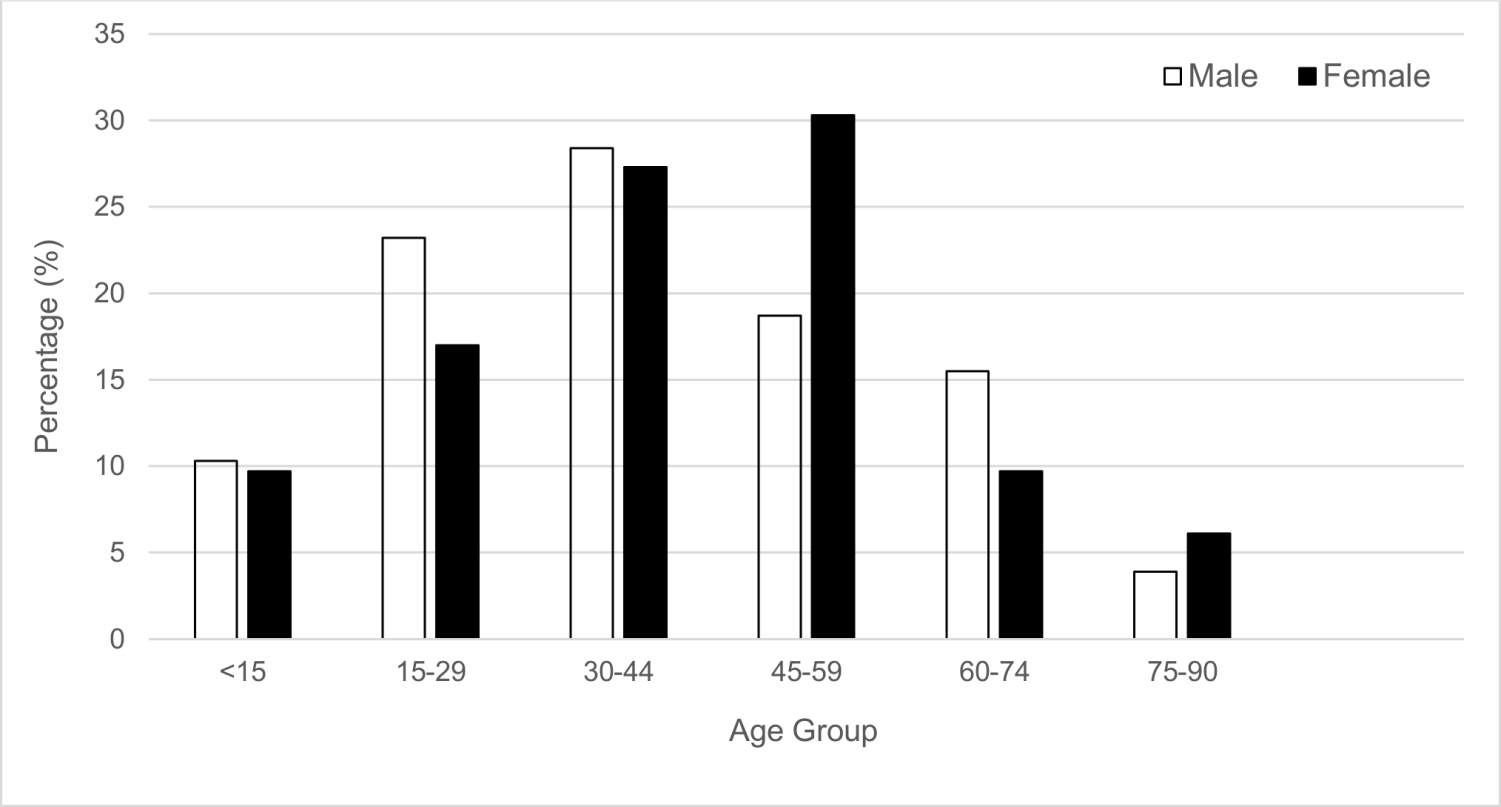
Mortality distribution across different age groups (of 15-year intervals) by gender.

There were no differences in gender (ᵡ^2^ = 0.82, df = 2, p = 0.664) and ethnic distributions (ᵡ^2^ = 10.86, df = 6, p = 0.093) across the age groups. Although the proportion of gender differed by only 2.4% in this cohort, there was a slight shift of mortality distribution to the right, where more females were affected with a peak at 45–59 age group (Fig 1). On the other hand, mortality among males peaked at a younger age group of 30–44 years.

West coast of Malaysia reported the highest dengue mortality cases, with more than one-third from Selangor (34.5%), followed by Johor (15.2%), Kuala Lumpur (8.7%), Perak (8.1%), Penang (6.5%), Malacca (5.3%), Negeri Sembilan (3.4%), Kedah (1.6%) and Perlis (0.3%). East coast contributed 10% to this study cohort, with 6.5% from Kelantan, 2.8% from Pahang and 0.6% from Terengganu, apart from the Borneo states, Sabah (3.4%) and Sarawak (3.1%). Only 38.9% of these cases documented they stayed at dengue at-risk areas, where there were more than one suspected dengue cases from the same area within the last one month prior to their symptoms onset.

### Time duration from first healthcare contact to death

Patients did seek treatment early with a median duration of one day from symptom onset to first healthcare contact (Table 2). Majority (84%) presented themselves to local healthcare facilities, either clinic or hospital, in public or private setting, within the first three days of their symptom onset, with 22.3% presented within the first 24 hours. About 14.1% sought treatment between four and seven days of their illness, with the remaining 1.9% presented late after one week.

**Table 2:** Median Duration from Symptom Onset to First Healthcare Contact, Hospital Admission, Death and Length of Stay for Dengue Deaths.

Duration (in days)	Overall	Children (<15 years)	Adults (15–59 years)	Elderly (≥60 years)
	median (25^th^ percentile, 75^th^ percentile)			
From symptom onset to first healthcare contact	1 (1, 3)	1 (0, 2.75)	2 (1, 3)	1 (1, 3)
From symptom onset to hospital admission	3 (2, 4)	3.5 (2, 4.75)	3 (2, 4)	3 (2, 4)
From hospital admission to ICU admission	0 (0, 1)	0 (0, 1.25)	1 (0, 1.25)	0 (0, 1.50)
Hospital stay	3 (1, 3)	2 (1, 5)	3 (2, 4)	3 (1, 4)
From symptom onset to death	6 (5, 8)	6 (4, 7)	6 (5, 8)	6 (5, 8)

Although all patients were subsequently admitted to hospital for further management, there was a slight delay of approximately two days between first medical contact and hospital admission. Up to half of them were admitted within first three day, and 44.7% between four and seven days of their illness. Majority (76.4%) were managed in intensive care units (ICU), with 51.8% of them warranted immediate transfer to ICU upon hospital admission for close monitoring and stabilization.

About 8.4% experienced rapid deterioration and died within 24 hours of admission. Meanwhile, 6.9% of deaths occurred early within first three days of illness, while 66.5% occurred between four and seven days which fell within the critical phase of dengue. The remaining quarter died more than a week after their symptoms onset. There was no significant difference in the duration from symptom onset to the first medical contact, hospital admission, ICU admission and death, across the age groups. Among 283 cases with location of deaths available, 89% occurred at ICU, 5% at emergency departments, 3.5% at high dependency units and 2.5% in general wards.

Among 11 children aged <15 years with their body mass indexes (BMI) available, 36.4% were obese (≥95^th^ centile of gender specific BMI-for-age), 36.4% were normal weight (5^th^-85^th^ centile) and the remaining underweight (<5^th^ centile). Out of the 114 adults, 26.3% were overweight (25–29.9kg/m^2^) with 42.1% were obese (≥30kg/m^2^). In the contrary, only 10% of the 20 elderly were obese and 30% were overweight, while more than half were of normal weight.

Nearly 90% of the elderly had underlying comorbid illness, but half of the children and 38.8% of the adult patients were free from any comorbid (ᵡ^2^ = 20.51, df = 2, p<0.001) (Table 3). Hypertension and diabetes mellitus were the most common comorbids in this cohort, especially among the elderly. In addition, dyslipidemia, heart diseases (ischaemic cardiomyopathy, heart failure and heart block), lung diseases (bronchial asthma and chronic obstructive pulmonary disease) and renal disease (chronic kidney disease) were more frequently observed among the elderly. Six adults had chronic liver disease, while another three had underlying malignancy.

**Table 3:** Presence of Co-morbids among Children, Adults and Elderly upon Hospital Admission.

	Overall	Children (<15 years)	Adults (15–59 years)	Elderly (≥60 years)	p-value
	n (%)				
**Presence of Co-morbid**	197 (61.6)	5 (15.6)	142 (61.2)	50 (89.3)	<0.001
**Co-morbidities**					
Diabetes mellitus	77 (24.1)	0	48 (20.7)	29 (51.8)	<0.001
Hypertension	78 (24.4)	1 (3.1)	42 (18.1)	35 (62.5)	<0.001
Dyslipidemia	11 (3.4)	0	6 (2.6)	5 (8.9)	0.056*
Heart disease	32 (10)	0	10 (4.3)	22 (39.3)	<0.001
Respiratory disease	20 (6.3)	3 (9.4)	11 (4.8)	6 (10.7)	0.152*
Renal disease	20 (6.3)	1 (3.1)	9 (3.9)	10 (17.9)	0.001*
Liver disease	6 (1.9)	0	6 (2.6)	0	0.641*
Carcinoma	3 (0.9)	0	2 (0.9)	1 (1.8)	0.622*

*Fisher’s exact test was performed instead of Pearson Chi-square test for this variable because more than 20% of expected counts were less than 5.

All patients presented with non-specific symptoms of febrile illness. Although 78.1% presented with at least one warning sign, one in every five patients with severe dengue in our cohort did not present with any warning sign (Table 4). Among those presented with dengue warning signs, up to 31.2% had only one warning signs which could be non-specific to dengue. The most common warning signs were lethargy or restlessness (42.5%) followed by persistent vomiting (38.6%) and laboratory markers indicating haemo-concentration (34.7%). Lethargy or restlessness (ᵡ^2^ = 8.509, df = 2, p = 0.014) were commoner in children while abdominal pain or tenderness was observed more frequently in the adults (ᵡ^2^ = 7.533, df = 2, p = 0.023).

**Table 4:** Presence of Warning Signs among Children, Adults and Elderly upon Hospital Admission.

	Overall	Children (<15 years)	Adults (15–59 years)	Elderly (≥60 years)	p-value
	n (%)				
**Presence of warning signs**					
Yes	250 (78.1)	25 (78.1)	181 (78)	44 (78.6)	0.996
No	70 (21.9)	7 (21.9)	51 (22)	12 (21.4)	
**Warning signs**					
Lethargy or restlessness					
Yes	136 (42.5)	20 (62.5)	88 (37.9)	28 (50)	0.014
No	184 (57.5)	12 (37.5)	144 (62.1)	28 (50)	
Persistent vomiting					
Yes	123 (38.6)	15 (46.9)	91 (39.2)	17 (30.9)	0.311
No	196 (61.4)	17 (53.1)	141 (60.8)	38 (69.1)	
Abdominal pain or tenderness					
Yes	106 (33.1)	8 (25)	87 (37.5)	11 (19.6)	0.023
No	214 (66.9)	24 (75)	145 (62.5)	45 (80.4)	
Mucosal bleeding					
Yes	46 (14.4)	3 (9.4)	38 (16.4)	5 (8.9)	0.252
No	274 (85.6)	29 (90.6)	194 (83.6)	51 (91.1)	
Clinical fluid accumulation (pleural effusion and/ or ascites)					
Yes	46 (14.4)	3 (9.4)	32 (13.8)	11 (19.6)	0.372
No	274 (85.6)	29 (90.6)	200 (86.2)	45 (80.4)	
Liver enlargement >2cm					
Yes	44 (13.8)	6 (18.8)	33 (14.2)	5 (8.9)	0.403
No	276 (86.2)	26 (81.2)	199 (85.8)	51 (91.1)	
Laboratory (increase in haematocrit with concurrent rapid decrease in platelet)					
Yes	111 (34.7)	8 (25)	86 (37.1)	17 (30.4)	0.306
No	209 (65.3)	23 (75)	146 (62.9)	39 (69.6)	

### Initial clinical diagnoses

Only 109 patients (34%), including a case of unknown age, were clinically diagnosed of dengue fever during their first contact with local healthcare facilities. Among those suspected of dengue, nearly half (48.6%) were diagnosed with dengue fever without warning signs, followed by dengue fever with warning signs (21.1%), severe dengue (17.8%) and DSS (11.5%). As compared to children, more adults and elderly presented with symptoms leading to early diagnosis dengue fever with warning signs, severe dengue and DSS. Non-specific viral febrile illness and upper respiratory tract infections were the commonest clinical impressions for patients across all the three age groups (Table 5). Two children presented with symptoms suggestive of meningoencephalitis. More adults and elderly presented with symptoms leading to the diagnoses of acute gastroenteritis and pneumonia.

**Table 5:** Initial Diagnoses at First Healthcare Contact.

Children (<15 years) (n = 32)	n	%
Acute pharyngitis and/ or tonsillitis	11	34.4
Non-specific (viral) febrile illness	10	31.3
Dengue fever without warning signs	2	6.3
Dengue fever with warning signs	2	6.3
Decompensated DSS	1	3.1
Meningoencephalitis with septicaemic shock	1	3.1
Status epilepticus with presumed meningitis and pneumonia	1	3.1
Acute gastroenteritis	1	3.1
Roseola fever	1	3.1
Acute bronchiolitis	1	3.1
**Adults (15–59 years)** (n = 232)		
Non-specific (viral) febrile illness	64	27.6
Dengue fever without warning signs	42	18.1
Acute pharyngitis and/ or tonsillitis	37	15.9
Dengue fever with warning signs	19	8.2
Severe dengue	14	6.0
Acute gastroenteritis	9	3.9
Compensated or decompensated DSS	7	3.0
Severe sepsis (infective diarrhoea, pneumonia)	7	3.0
Clinical leptospirosis	3	1.3
Urinary tract infection	2	0.9
Acute appendicitis	2	0.9
Bodyache and/ or headache	2	0.9
Labial abscess	1	0.4
Nephrolithiasis	1	0.4
Gastritis	1	0.4
Presumed measles	1	0.4
Presumed typhus	1	0.4
**Elderly (≥60 years)** (n = 56)		
Non-specific (viral) febrile illness	12	21.4
Dengue fever without warning signs	9	16.0
Severe dengue	5	8.9
Compensated or decompensated DSS	5	8.9
Community acquired pneumonia with/ without septicaemic shock	5	8.9
Acute pharyngitis and/ or tonsillitis	3	5.3
Dengue fever with warning signs	2	3.5
Headache	2	3.5
Acute gastroenteritis	1	1.7
Severe sepsis with multiorgan failure	1	1.7
Advanced carcinoma rectum	1	1.7
Acute stroke	1	1.7
Urinary tract infection	1	1.7
Acute upper gastrointestinal bleeding	1	1.7
Indigestion	1	1.7

Details on diagnoses were missing in 1 patient (3.1%) for the children group, 19 for the adults (8.2%) and 7 for the elderly (12.5%).

During the clinical assessment upon hospital admission, the index of suspicion for dengue had increased to 73.6%, among which 46.8% were diagnosed as severe dengue or DSS, 28.7% as dengue fever with warning signs and 24.5% were dengue infection without warning signs. Approximately a quarter of cases were remained diagnosed as non-dengue conditions, with a small proportion (2.8%) were treated as severe sepsis (Table 6). Some patients first sought medical care at hospitals leading to immediate admission, hence giving rise to overlapping of clinical diagnoses in Tables 5 and 6.

**Table 6:** Diagnoses upon Hospital Admission.

Children (<15 years) (n = 32)	n	%
Severe dengue/ DSS	8	25.0
Compensated or decompensated DSS	6	18.8
Dengue fever without warning signs	3	9.4
Acute pharyngitis and/ or tonsillitis	3	9.4
Meningoencephalitis with septicaemic shock	3	9.4
Non-specific (viral) febrile illness	2	6.3
Dengue fever with warning signs	1	3.1
Acute gastroenteritis	1	3.1
Neonatal sepsis	1	3.1
Status epilepticus with pneumonia	1	3.1
Presumed malaria	1	3.1
**Adults (15–59 years)** (n = 232)		
Dengue fever with warning signs	59	25.4
Dengue fever without warning signs	46	19.8
Compensated or decompensated DSS	40	17.2
Severe dengue	39	16.8
Non-specific (viral) febrile illness	10	4.3
Clinical leptospirosis	8	3.4
Severe sepsis (pneumonia, urosepsis)	7	3.0
Diabetic ketoacidosis	3	1.3
Acute upper gastrointestinal bleeding	2	0.9
Acute appendicitis	2	0.9
Acute gastroenteritis	2	0.9
Acute hepatitis or acute liver failure	2	0.9
Obstructive sleep apnoea with right heart failure	1	0.4
Anaemia secondary to chronic illness	1	0.4
Community acquired pneumonia	1	0.4
**Elderly (≥60 years)** (n = 56)		
Dengue fever without warning signs	9	16.1
Severe dengue	9	16.1
Compensated or decompensated DSS	8	14.3
Dengue fever with warning signs	7	12.5
Community acquired pneumonia with/ without septicaemic shock	3	5.4
Acute gastroenteritis	2	3.6
Acute upper gastrointestinal bleeding	2	3.6
Non-specific (viral) febrile illness	1	1.7
Acute exacerbation of chronic obstructive pulmonary disease	1	1.7
Severe sepsis with multiorgan failure	1	1.7
Acute stroke with aspiration pneumonia	1	1.7
Intestinal obstruction	1	1.7
Seizure	1	1.7
Septic colitis	1	1.7
Clinical leptospirosis	1	1.7

Details on diagnoses were missing in 2 patient (6.3%) for the children group, 9 for the adults (3.9%) and 8 for the elderly (14.3%).

### Dengue serology

Among 285 cases (69.9%) had their sera samples tested reactive for dengue NS1 antigen, two-third had their tests performed between days three and five from their symptoms onset. However, about 17.3% were tested reactive within 48 hours of their illness, while 14.2% between days 6 and 10, with the remaining 1.8% detected between days 11 and 16.

IgM was detected in the sera of 196 patients (60.9%). Nearly 20% had their dengue IgM detected within 48 hours of their symptoms onset, 44.4% between days three and five, 32.1% between days 6 and 10, and 3.6% beyond day 10 of illness. One-third of the patients were immunoglobulin G (IgG) positive. Two patients with NS1 and IgM seronegative had their diagnoses confirmed with PCR.

### Clinical progression to severe dengue

As the disease progressed, all patients fulfilled at least one of the components for the severe dengue. Nearly 80% had severe organ involvement with 65% experienced severe plasma leakage and a third suffered from severe bleeding (Table 7). More adults had severe plasma leakage, as compared to children and elderly (ᵡ^2^ = 8.016, df = 2, p = 0.018). Neurological involvement in severe dengue were commoner in children (ᵡ^2^ = 9.392, df = 2, p = 0.009). On the other hand, heart involvement were observed more frequently among elderly (ᵡ^2^ = 7.802, df = 2, p = 0.020), especially those with underlying heart disease.

**Table 7:** Criteria of Severe Dengue Based on Age Groups.

	Overall	Children (<15 years)	Adults (15–59 years)	Elderly (≥60 years)	p-value
	n (%)				
**Severe plasma leakage**					
Yes	211 (65.3)	20 (62.5)	162 (69.8)	28 (50.0)	0.018
No	111 (34.4)	12 (37.5)	70 (30.2)	28 (50.0)	
Respiratory distress due to plasma leakage					
Yes	95 (29.4)	9 (28.1)	73 (31.5)	13 (23.2)	0.469
No	227 (70.3)	23 (71.9)	159 (68.5)	43 (76.8)	
**Severe bleeding**					
Yes	112 (34.7)	12 (37.5)	82 (35.3)	17 (30.4)	0.734
No	210 (65.0)	20 (62.5)	150 (64.7)	39 (69.6)	
**Severe organ involvement**					
Yes	257 (79.8)	26 (81.2)	183 (78.9)	46 (82.1)	0.839
No	65 (20.2)	6 (18.8)	49 (21.1)	10 (17.9)	
Liver					
Yes	194 (60.2)	20 (62.5)	143 (61.6)	30 (53.6)	0.523
No	128 (39.8)	12 (37.5)	89 (38.4)	26 (46.4)	
Kidneys					
Yes	139 (43.2)	15 (46.9)	93 (40.1)	31 (55.4)	0.108
No	183 (56.8)	17 (53.1)	139 (59.9)	25 (44.6)	
Central nervous system					
Yes	82 (25.5)	15 (46.9)	54 (23.3)	11 (19.6)	0.009
No	240 (74.5)	17 (53.1)	178 (76.7)	45 (80.4)	
Heart					
Yes	44 (13.7)	5 (15.6)	25 (10.8)	14 (25.0)	0.020
No	278 (86.3)	27 (84.4)	207 (89.2)	42 (75.0)	

Among 140 patients with bleeding tendencies, 112 (80%) experienced severe bleeding. Acute gastrointestinal bleeding, involving either upper and/or lower gastrointestinal tract, was the commonest haemorrhagic complications (37.8%), followed by pulmonary haemorrhage (11.4%), occult bleeding (5%), intracranial bleeding (3.6%) and vaginal bleeding (3.6%). Hematuria (0.7%), intraabdominal haemorrhage (0.7%), and post-partum haemorrhage after Caesarean section (0.7%) were the rare complications reported. Ten patients developed disseminated intravascular coagulopathy. Other sites with milder bleeding included mucosa (gum and nasal cavity) (11.4%), venous puncture sites (4.3), and abdominal wall (0.7%).

In our cohort, 46.6% of the patients received transfusion of blood products. Seventy-eight patients (24.1%) required emergency dialysis for acute renal failure, with only nine of them had pre-existing renal disease. Nearly half (49.2%) of them required mechanical ventilatory support. Six patients had their bone marrow biopsy showing reactive haemophagocytosis activity.

More than half (55.7%) of these patients had DSS. More adults (59.9%) suffered from DSS, as compared to children (50%) and the elderly (42.9%) (ᵡ^2^ = 5.833, df = 2, p = 0.054).

Dengue IgG positivity was associated with higher proportion of severe plasma leakage (ᵡ^2^ = 6.889, df = 2, p = 0.032), but not DSS (ᵡ^2^ = 3.015, df = 2, p = 0.221), severe bleeding (ᵡ^2^ = 0.530, df = 2, p = 0.767) and severe organ involvement (ᵡ^2^ = 2.451, df = 2, p = 0.294).

### Causes of dengue death

**Fig 2:**
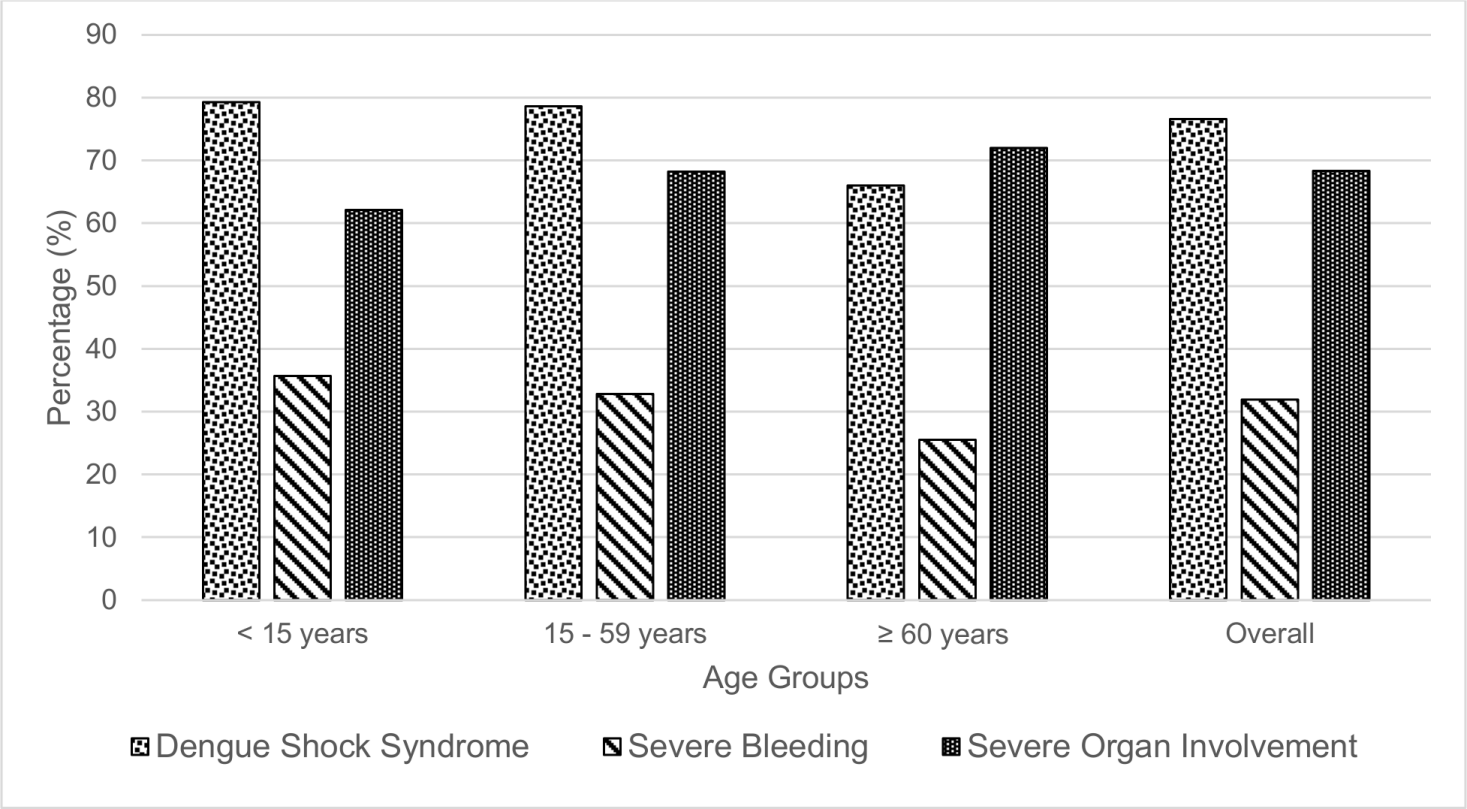
Causes of dengue death among children, adults and elderly.

Classifying the cause of death for each case remained challenging, as so for some patients, more than one cause might have contributed to their deaths. Overall, DSS was the most common cause of death among our cohort (72.9%), followed by severe organ dysfunction (69%) and severe bleeding (29.7%) (Fig 2). There was no significant difference in causes of death across different age groups in our cohort. Approximately half (51.9%) of the mortality were contributed by combinational causes, and 16.6% died of DSS complicated with both severe bleeding and severe organ involvement.

## Discussion

Dengue infection is associated with severe disease, and deaths occur despite supportive management. This is the first study at national scale to investigate epidemiology, clinical characteristics and causes of death of fatal dengue cases in Malaysia to date. This study also provides additional information to fill in the gap of understanding in term of the warning sign manifestations, clinical diagnoses at initial presentation, severe dengue and causes of death across three different age groups.

Over the recent years, there is a shift of the infection from affecting primarily children to adults [7, 8, 10]. The Malaysian Cohort Study in 2008 reported 91.6% of adults aged 35–74 years were seropositive for dengue IgG [11]. This rate increased with age, and reached nearly 100% by 60 years of age [9]. The mean age of the fatal cases in our cohort was 40.7 ±19.3 years, with 72.5% of them were from working age group of 15–59 years. We postulate the higher proportion of secondary dengue infection among adults which might have contributed partly to the age shift in mortality. Among 112 IgG seropositive patients in this cohort, 73% were from the adult group and 19% from the elderly. Secondary dengue infection has been associated with severe outcome of dengue via antibody-dependent enhancement [11] and T-cell original antigenic sin [12].

Some studies have observed that early care-seeking behaviour may determine the possibility of receiving optimum treatment and thereby avoiding fatal outcomes [13–15], and gender might affect the care-seeking behaviour patterns [16]. However, we were unable to demonstrate any association between the care-seeking behaviour pattern and gender, as well as age groups in this cohort. Majority of our patients sought treatment early regardless of gender and age groups. The median duration from onset of illness to first healthcare facilities visit was 1 day for children and elderly while 2 days for adults. Patients in this cohort were hospitalized early by a median duration of illness of 3 days as compared mean of 4.7 days as reported by Sam SS *et al*. [15]. Due to rapidly progressive clinical deterioration, they were also admitted to ICU earlier on a median of 3 days of illness in contrast to a mean of 5.6 days as reported in Singapore [13], but similar to 3.75 days in Cuba [17].

Dengue death was observed more frequently among patients who had sought care after the fourth or fifth day of fever, compared to those who sought care during the first three days of fever [17, 18]. However, 84% in this study was found to seek treatment early within first 3 days of illness. The slight delay of median 2 days between duration of fever onset to first visit to health care facilities and subsequent hospital admission reflected not only the challenges in identifying dengue in the early course of disease, but the dynamic progression of the infection itself, as 21.9% did not experience any warning signs at initial presentation. This observation is inconsistent with the expectation that timely treatment following early identification of the illness might have resulted in better survival outcome. In fact, only 35% of adults and 37.5% of elderly were clinically diagnosed with dengue at their first presentation to healthcare facilities. This was consistent with study by Jenny Low GH *et al*., who reported early clinical diagnosis based on WHO classification is more difficult in older adults [19]. Despite WHO had developed a set of guidelines to aid in diagnosis of dengue infection and disease classification, it remains challenging to differentiate dengue infection clinically from other causes of febrile illness, especially during the early phase of illness [20]. It may also be contributed by the atypical presentations among adults age >18 years as the frequency of symptoms and signs reported by adult population in accordance to the WHO classification schemes (1997 and 2009) reduced significantly with increasing age [19].

Using the WHO recommendations for BMI, the national prevalence of overweight and obesity among adults aged 18 years and above were 33.6% and 19.5% respectively [21]. Besides, the National Health Morbidity Survey III reported 19.9% of Malaysian children aged 7–12 years were overweight [22]. The fatal cases in our cohort appeared to have higher proportion of overweight and obesity as compared the national prevalence.

The presence of co-morbidities and other chronic illnesses might contribute to increased dengue mortality in adult population [13, 23, 24]. In this review, 64.9% had underlying co-morbid illness. Nonetheless, 38.8% adults and 10.7% elderly were healthy individuals without any co-morbids. This observation highlighted that underlying co-morbidities might not be the main factor contributed to the dengue mortality in our cohort.

This study also reviewed the warning signs of fatal dengue cases. It has been suggested that many warning signs are uncommon on their own, but lethargy or restlessness, abdominal pain or tenderness and mucosal bleeding were the three commonest occurring before the development of severe dengue [25]. This review reported the similar finding that lethargy was the commonest warning sign observed in the cohort. However, none of the warning signs presented in more than half of these fatal cases. Interestingly, 21.9% did not have any warning signs upon hospital admission. This finding was lower compared to a study by Thein TL *et al*. where 58% of their cohort did not have any warning signs prior to development of severe dengue [25]. This gap was probably attributed to the different study populations whereby our study focused only on fatal cases and excluded survivors of severe dengue. While lethargy or restlessness was commoner among children and abdominal pain or tenderness was observed more often in adults, there was no difference in other warning signs at presentation across the three age groups. This is in accordance to abdominal pain and lethargy being well-established warning signs which contributed to severe dengue and dengue deaths [25–27], but there is limited literature illustrating the association of warning signs with different age groups.

Being age >40 years was an independent risk factor for the development of severe dengue [28]. More adults in this review developed severe plasma leakage as compared to children and elderly. There is anecdotal evidence suggesting both hepatic dysfunction and bleeding manifestations are more common in older age groups [13, 29, 30], but not in our study population. In contrast, we observed a higher proportion of heart involvement among the elderly whom with underlying heart diseases, which may have been exacerbated by the effects of acute infections. Neurological involvement was observed more frequently among children, which is consistent with a review by Puccioni-Sohler M *et al*. [31], yet the neuropathogenesis of dengue infection remains poorly understood. Some proposed possible mechanisms for neurological involvement are direct viral infection of central nervous system, autoimmune reaction, metabolic and hemorrhagic disturbances [32].

In this study, we attempted to classify the primary cause of deaths to DSS, severe bleeding and/or severe organ dysfunction, based on the causes of death retrospectively determined. DSS was the commonest cause of death (72.9%) in our cohort, followed by severe organ involvement (69%) and severe bleeding (29.7%). Our findings are similar to another review on fatal cases from Singapore which reported shock as the commonest cause of death followed by organ impairment (71.4% with acute renal impairment, 57.1% with impaired consciousness and 53.6% with severe hepatitis) [33]. There was slightly higher proportion of deaths attributable DSS alone among children as compared to adults and elderly. Gamble J *et al*. reported children are more prone to develop hypovolaemic shock as compared to adults in any conditions characterized by increased microvascular permeability because they have a larger microvascular surface area per unit volume of skeletal muscle, and the proportion of the developing vessels is greater during development [34]. These developing microvessels are known to be more permeable to water and plasma proteins than when they mature [34].

The strength of this study lies in our attempt to review all dengue-related mortality cases reported countrywide between 2013 and 2014, according to different age groups. Each case was reviewed extensively and causes of death were retrospectively determined and revised by an independent panel of consultant physicians at dengue mortality meeting. In our attempt to report the basic epidemiological characteristics of these fatal cases and their clinical course of their illness from initial presentation until deaths ensued, we recognize the lack of access to the individual case record imposed a quality limitation to clinical data collection. Although we attempted to collect relevant data in details, we acknowledge the possibility of recall bias by the managing team, information and reporting biases during documentation process. The absence of detailed information with regards to organ involvement in organ failure and those resulted in death, as well as management plan for patients during their illness have led to limited analyses in this study. We have identified some but not all the co-morbidities, especially in the elderly as limited by the retrospective nature of this review. We were unable to assess the risk factors for severe dengue and associated mortality due to lack of a control group. All patients in this study were hospitalized and the current surveillance system is yet to be strengthened to capture dengue-related deaths occur at home or as out-patients.

Dengue is a dynamic disease which its clinical progression can evolve beyond anticipation. In Malaysia, majority of the dengue-related deaths occurred in adults of working age group between 15–59 years. Although many have sought treatment early, the index of suspicion for diagnosing dengue remained low at their initial presentation. More than 60% subsequently succumbed to DSS and severe organ involvement refractory to aggressive intervention.

## Supporting Information

S1 Fig Case Report Form of dengue mortality cases. (PDF)

S2 Fig STROBE checklist. (DOCX)
