# Supplementary material for: A Two-Year Review on Epidemiology and Clinical Characteristics of Dengue Deaths in Malaysia, 2013-2014
Source: PLoS Negl Trop Dis. 2016 May 20;10(5):e0004575. doi: 10.1371/journal.pntd.0004575 (PMC4874788; doi:10.1371/journal.pntd.0004575)
Supplement: S1 Fig — (PDF) [file pntd.0004575.s001.pdf]

INVESTIGATION OF DENGUE DEATH

DEMOGRAPHICS

Name:

IC: Age: Sex:

Race:

Name of Hospital:

Dengue at-risk area ☐ No ☐ Yes

Death location ☐ A&E ☐ Ward ☐ HDU ☐ ICU

First health care contact

First visit to healthcare facilities Date: Time:

Hospital Admission via

Date of Registration A&E: Time:

Time seen by doctor: Seen by:

First reviewed by Specialist: Date: Time:

Admission to ward/ ICU/ HDU

Date of Admission: Time:

Time seen by doctor: Seen by: ☐ Houseman ☐ Medical Officer

Severity Of Illness On Admission

Date of onset of fever:

Body weight (kg): Height (cm): BMI:

Warning Signs (Tick √ where appropriate):

- ☐ Abdominal pains and tenderness
- ☐ Persistent vomiting
- ☐ Clinical fluid accumulation
- ☐ Mucosal bleed
- ☐ Lethargy, restlessness
- ☐ Liver enlargement > 2cm
- ☐ Laboratory: increase in HCT concurrent with rapid decrease in platelet count

Initial Diagnosis at A&E / OPD:

- ☐ Dengue without warning signs
- ☐ Dengue with warning signs
- ☐ Severe Dengue
- ☐ Others (please specify):

DENGUE DIAGNOSTIC RESULTS

NS1 Ag reactive:

Dengue IgM reactive: Date:

Dengue IgG reactive: Date:

****CRITERIA FOR SEVERE DENGUE:**

(Tick √ where appropriate)

Severe plasma leakage

- ☐ Shock (DSS)
- ☐ Fluid accumulation with respiratory distress

☐ **Severe bleeding (as evaluated by clinician)** Specify

Severe organ involvement

- ☐ Liver Specify
- ☐ CNS Specify
- ☐ Heart and other organs Specify
- ☐ Other organs Specify

Co morbid conditions

☐ Immunocompromised (e.g. Leukemia / HIV Positive / Patient on steroid / Chemotherapy)

☐ Renal Failure

☐ Obesity (based on BMI (adult) or NCHS chart [children])

☐ Pregnancy

☐ Heart Ailments

☐ Others (specify)

Comment: …….

……

DEATH

Date of Death: Time:

Immediate cause of death:

- ☐ Dengue Shock Syndrome
- ☐ Severe Bleeding Specify
- ☐ End / Multi-organ failure Specify
- ☐ Others Specify

Comment:……

………
